# Washable Colorimetric Nanofiber Nonwoven for Ammonia Gas Detection

**DOI:** 10.3390/polym12071585

**Published:** 2020-07-16

**Authors:** Hyun Ju Oh, Byeong Jin Yeang, Young Ki Park, Hyun Jung Choi, Jong H. Kim, Young Sik Kang, Younghwan Bae, Jung Yeon Kim, Seung Ju Lim, Woosung Lee, Wan-Gyu Hahm

**Affiliations:** 1 Advanced Textile R&D Department, Korea Institute of Industrial Technology, Ansan 15588, Korea; hjoh33@kitech.re.kr (H.J.O.); yeang777@kitech.re.kr (B.J.Y.); yhwanee@kitech.re.kr (Y.B.); cobalt98@kitech.re.kr (J.Y.K.); 2 Test-Bed Research Center, Korea Dyeing & Finishing Technology Institute (DYETEC), Daegu 41706, Korea; parkyk@dyetec.or.kr; 3 R&D Center, PNC Labs Inc., Osan 18103, Korea; hyunjung.choi@pnclabs.co.kr; 4 Department of Molecular Science and Technology, Ajou University, Suwon 16499, Korea; jonghkim@ajou.ac.kr; 5 Research Institute, Dissol Co., Ltd., Jeonju 54853, Korea; kys@dissol.kr; 6 Department of Advanced Materials Engineering for Information & Electronics, Kyung Hee University, Yongin 17104, Korea

**Keywords:** ammonia gas, colorimetric nanofiber sensor, meta-aramid nanofiber, gas detection

## Abstract

The colorimetric sensor is a facile, cost-effective, and non-power-operated green energy material for gas detection. In this study, the colorimetric sensing property of a meta-aramid/dye 3 nanofiber sensor for ammonia (NH_3_) gas detection was investigated. This colorimetric sensor was prepared using various dye 3 concentrations via electrospinning. Morphological, thermal, structural, and mechanical analyses of the sensor were carried out by field-emission scanning electron microscopy, thermogravimetric analysis, Fourier-transform infrared spectroscopy, and a universal testing machine, respectively. A homemade computer color matching machine connected with a gas flow device characterized the response of the meta-aramid/dye 3 nanofiber colorimetric sensor to various exposure levels of NH_3_ gas. From the results, we confirmed that this colorimetric green energy sensor could detect ammonia gas in the concentration of 1–10 ppm with a sensing response time of 10 s at room temperature. After washing with laundry detergent for 30 min, the colorimetric sensors still exhibited sensing property and reversibility.

## 1. Introduction

Ammonia has been widely used as a catalyst or reagent in various industries such as agriculture, living environments, medical treatments, and other industrial applications [1]. Ammonia gas (NH_3_) is colorless, explosive toxic, and has a pungent smell. The NH_3_ concentration limit set by the occupational safety and health administration is 25 ppm and people feel uncomfortable above this level due to odor and irritation. Furthermore, if the concentration of NH_3_ increased up to 300 ppm, it may lead to death [2,3,4,5]. Thus, it is important to continuously detect and monitor the leakage of NH_3_ to guarantee the health and safety on industrial sites and in daily life.

Over the past few decades, various types of gas sensors have been developed based on semiconductors, optical fibers, acoustic waves, and other technologies [6,7,8,9]. Recently, many efforts have been made to make the sensors smaller, more sensitive, provide a visual response, easy to handle, personal, and wearable. However, most of these detections require expensive instruments, complex techniques, and electrical power, and they are expensive [10]. In this respect, colorimetric sensors are of great interest due to their simplicity, easy fabrication, low-cost, visual response, and ease of interpretation without any power consumption [11,12,13,14,15,16]. The working principle of such colorimetric sensors is based on color changes by various mechanisms such as photochromism (light), electrochromism (oxidation/reduction), thermochromism (heat), solvatochromism (solvent polarity), ionochromism (ions), and halochromism (pH) [10]. Up until now, numerous colorimetric sensors in the form of films [3,17], aerogel [4,18], nanofibers [19], and fabrics [20] were investigated to detect various hazardous gases such as ammonia [2,3,4,21], phosgene [22], hydrogen sulfide [23], and volatile organic compounds (VOCs) [18]. Khattab et al. reported that the cotton biosensor fabric coated with alginate capsulated with the protonated tricyanofuran hydrazine anion detected the urease enzymatic activity and showed the color change from light yellow to purple [24]. Kim et al. introduced that the M13 bacteriophage-based multiarray colorimetric sensor, which can detect drug contaminants such as hormone drugs (estrogen) and antibiotics [16].

Nanofibers have attracted great attention in advanced sensor design in recent years. Nanofibers offer many advantages for gas sensing such as high specific area, high volume of small pores, and good inter-pore connectivity resulting in a fast response time [25,26,27]. Furthermore, various surface morphologies of nanofibers such as net, porous, and open structure can be easily produced by modifying the solution conditions and manufacturing process parameters [10,28]. Ding et al. used this approach to develop the colorimetric nylon 6 nanofiber/nets (NFN) membranes impregnated with methyl yellow colorant for detecting formaldehyde gas [29]. This study showed that the nylon 6 NFN strip successfully changed its color from yellow to red after exposure to formaldehyde gas with a low detection limits of 50 ppb, and these nano-nets could enhance the interconnectivity, surface area, and hence the diffusion of analytes into the membranes. Kim et al. used bromocresol green (BCG) to produce an NH_3_ detectable polyacrylonitrile (PAN) nanofiber colorimetric sensor. This sensor showed fast detection time (<1 min at 25 ppm) and excellent selectivity toward common volatile organic solvents (VOCs) [30]. However, at the current stage, colorimetric sensors still have some problems to overcome such as sensitivity, high cost, reversibility, reusability, and washability for their safer use in our daily life [31].

**Figure 1:**
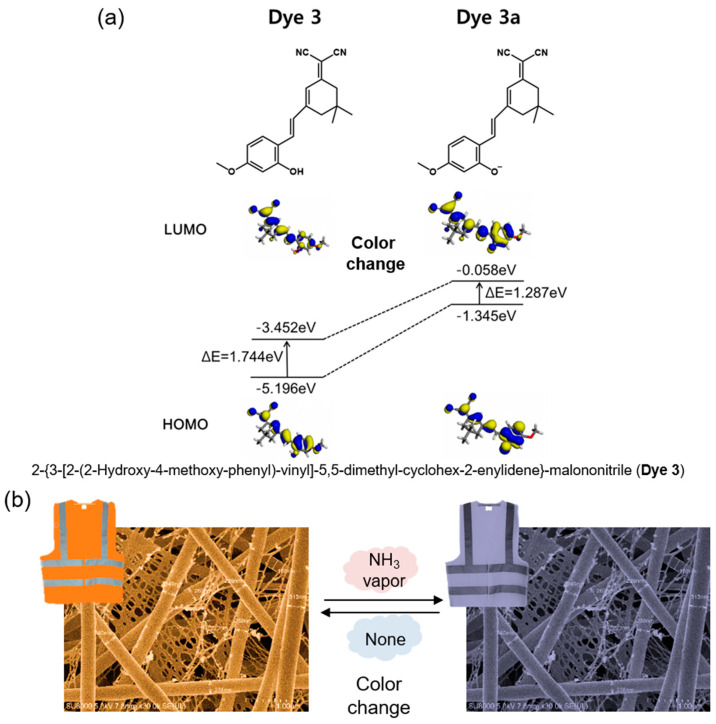
(**a**) Electron distribution of the HOMO and LUMO energy levels of dye 3 and dye 3a [32] and (**b**) a scheme depicting the color change of the colorimetric nanofiber sensor when exposed to NH_3_ gas.

In this study, the sensing property of a fabricated colorimetric meta-aramid/dye 3 nanofiber NH_3_ gas sensor was investigated. These textile sensor specimens containing various dye 3 concentrations were fabricated using electrospinning. The surface morphology of the meta-aramid/dye 3 nanofiber sensor specimens was observed by field emission scanning electron microscopy (FE-SEM). A homemade gas tester consisting of a computer color matching system connected with a gas flow device analyzed the colorimetric sensing property of the sensor. When dye 3 was exposed to ammonia gas, deprotonation of dye 3 shifted the absorption band bathochromically as shown in Figure 1a [32] and was accompanied by a change in the color of the sensor from orange to dark brown (Figure 1b). After washing with laundry detergent for 30 min, the colorimetric sensing test was performed again to confirm the durability and reusability of the colorimetric sensor.

## 2. Materials and Methods

### 2.1. Synthesis of Meta-Aramid

In this work, the meta-aramid was synthesized by a well-known procedure described in the literature and our previous paper [33,34] that used an equal molar ratio of m-phenylene diamine (MPD; P23954, Sigma Aldrich, St. Louis, MO, USA) and 1,3-isophthaloyl chloride (IPA; I19403, Sigma Aldrich, St. Louis, MO, USA) in DMAc (D0484, Samchun, Seoul, Korea). During the reaction, HCl was formed and was neutralized by the addition of Ca(OH). After neutralization, a CaCl_2_ salt byproduct was produced, which was used for the electrospinning process without further purification. After polymerization, the meta-aramid with an average molecular weight of 381,000 g/mol (DMSO, GPC) was obtained [34,35].

### 2.2. Synthesis of Dye 3

**Figure 2:**
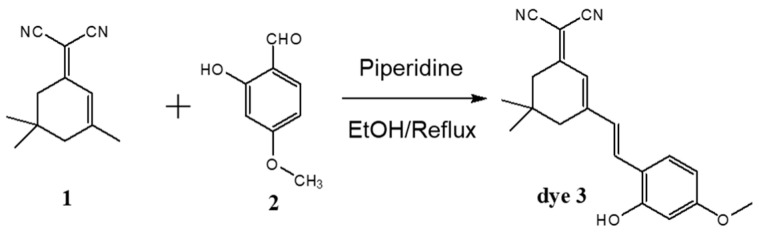
The synthetic route for dye 3.

The D-π-A dye (2-{3-[2-(2-hydroxy-4-methoxy-phenyl)-vinyl]-5,5-dimethyl-cyclohex-2-enylidene}-malononitrile, dye 3) for ammonia gas detection was also synthesized as described in our previous study [32]. An equal molar ratio of 2-(3,5,5-trimethylcyclohex-2-enylidene)malononitrile and 2-hydroxy-4-methoxybenzaldehyde was dissolved in ethanol. Piperidine was titrated to this solution and it refluxed for 7 h. Next, it was filtered and purified (yield = 60%). The synthetic route of dye 3 is shown in Figure 2.

### 2.3. Fabrication of Meta-Aramid/Dye 3 Colorimetric Nanofiber Sensors

The meta-aramid was dissolved in DMAc at 60 °C to prepare a meta-aramid solution (14%, *w*/*w*). Dye 3 was added to this solution at a concentration of 1, 5, and 10 wt %; the specimen labels are listed in the Table 1. The meta-aramid/dye 3 nanofibers were fabricated by electrospinning. The homogeneous solution was inserted into a plastic syringe and injected through a metal needle (25G) connected to a high-voltage generator (NNC-HV30, NanoNC, Seoul, Korea). The solution was fed at a rate of 10 μL/min by using a syringe pump (EP100, NanoNC, Seoul, Korea). The applied voltage was 15 kV, and the distance between the metal tip and collector was 10 cm. The orange colored meta-aramid/dye 3 nanofiber mat was collected on the surface of a release paper loaded on a collector rotating at 300 rpm. The mat was then dried at 50 °C under vacuum for 24 h.

### 2.4. Characterization

The surface morphology of the meta-aramid/dye 3 nanofibers was observed by FE-SEM (SU8010, Hitachi Co., Tokyo, Japan) with an acceleration voltage of 10 kV after sputter coating with platinum (Pt). The contact angle and porosity of meta-aramid/dye 3 nanofiber mats were characterized using a drop shape analysis system (DSA100, KRUSS, Hamburg, Germany) with distilled water, and mercury porosimetry (Auto pore IV 9500, Micromeritics, Norcross, GA, USA) according to ISO 15901-1, respectively. The average diameter of meta-aramid/dye 3 nanofibers was measured for at least 10 specimens. Thermal stability of the nanofibers was characterized by thermogravimetric analysis (TGA; Q500, TA Instruments, New castle, DE, USA) in N_2_ gas at 10 °C/min heating rate. The surface bonding configurations of the meta-aramid/dye 3 nanofiber specimens were analyzed from the Fourier-transform infrared spectroscopy (FT-IR) profiles using a Thermo Nicolet iS50 (Thermo Fisher Scientific, Waltham, MA, USA). The scan range was 650–4000 cm^−1^. The Brunauer–Emmett–Teller (BET) surface area was evaluated by a surface area analyzer (ASAP 2010, Micromeritics, Norcross, GA, USA) using N_2_ adsorption–desorption according to ISO15901-2. The tensile strength of meta-aramid/DM nanofiber specimens was measured by a universal material testing machine (Model 5567, Instron, Norwood, MA, USA) based on the ASTM standard D 638. The gauge length and crosshead speed were 3.18 mm and 1 mm/min, respectively. The test was conducted on at least 10 specimens and the average value was reported.

### 2.5. Gas Test of the Colorimetric Nanofiber Sensor

**Figure 3:**
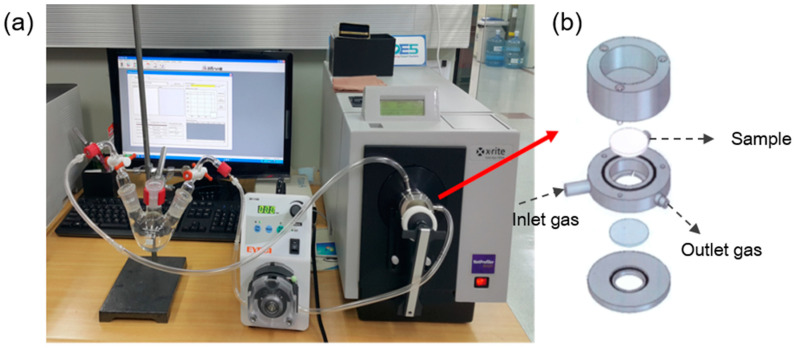
Color measurement system connected with a gas flow device [20]: (**a**) picture of our entire system and (**b**) schematic diagram of the chamber with a loaded sample.

The sensing ability of the meta-aramid/dye 3 nanofiber textile was characterized by the gas test system as described in our previous literature [20]. The gas test system designed to measure the real-time color change of the fabricated textile sensors when exposed to gas is shown in Figure 3. The sensors were placed in a stainless-steel chamber and NH_3_ at a controlled concentration was circulated through the Teflon tube connected to the chamber. Since the internal volume of the entire system was measured, the concentration of the gas could be controlled by the amount of liquid injected into a three-neck round bottom flask. A heat gun was used to evaporate the injected liquid in the flask, and the gas was circulated in the entire system using a peristaltic pump. After exposure to the gas, the dynamic color change of the textile sensor specimens was measured by a color-eye 7000A spectrophotometer (X-rite, Grand Rapids, MI, USA).

The color change (ΔE) and surface color strength (K/S) were calculated using the following equation [36]:

(1) $$\Delta E=\sqrt{{\left(\Delta L^{*}\right)}^{2}+{\left(\Delta a^{*}\right)}^{2}+{\left(\Delta b^{*}\right)}^{2\;}}$$

where L* is lightness, a* represents red/green component, and b* represents yellow/blue component. NH_3_ gas was used at a concentration of 1–10 ppm, and the color change was measured every 10 s for 5 min.

### 2.6. Color Fastness Test

The fastness test of meta-aramid/dye 3 nanofiber sensor specimens with various dye 3 concentrations was performed according to method A of standard ISO 105C10. The meta-aramid/dye 3 nanofibers were washed for 30 min at the temperature of 40 °C. SDCE standard soap (5 g/L; SDCE Type 1, SDC Enterprises Ltd., Bradford, UK) was used, and the bath ratio was 50:1. The sample dimension was 100 × 40 mm and the nanofiber was covered with the ISO DW type-multi fiber fabric (MFF 10A, Testfabrics Korea, Inc., Ansan, Korea).

## 3. Results and Discussion

**Figure 4:**
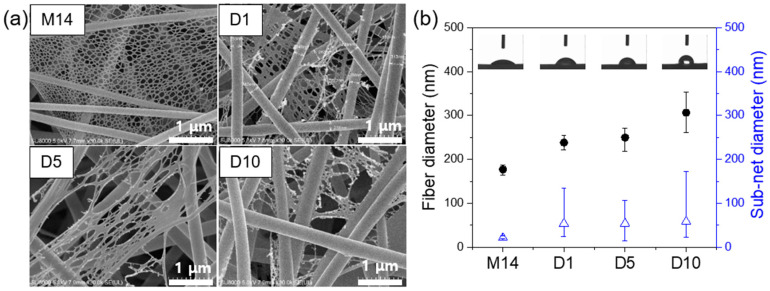
(**a**) FE-SEM images of the electrospun meta-aramid mats with various dye 3 concentrations and (**b**) fiber and sub-net size distribution of as-prepared nanofiber mats.

The surface morphologies of as-prepared meta-aramid and meta-aramid/dye 3 nanofibers are shown in Figure 4a. The average diameter of the pristine meta-aramid nanofibers was approximately 177 ± 11 nm. The specimen clearly exhibited the NFN structure in the meta-aramid membranes. As the dye 3 concentration increased, the average diameter of the meta-aramid nanofibers significantly increased (306 ± 45 nm). With the introduction of dye 3, the sub-net structure showed a lush shape, such as merging or breaking ends. From the water contact angle measurements, it was confirmed that the wettability of meta-aramid nanofiber mat containing the hydrophobic dye 3 indicator was significantly decreased than that of the pristine nanofiber mat. The water contact angle of meta-aramid nanofiber was significantly increased from 44.0 to 112.0° with an increase in the amount of dye 3. In addition, the porosity of meta-aramid/dye 3 nanofiber mat was slightly decreased than that of pristine nanofiber mat, and it was also affected by an increase in the diameter of nanofiber and merged sub nano-net. The literature reports several causes of the NFN formation such as phase separation of charged droplets, ion-initiated splitting up, and inter-molecular hydrogen bonding [37]. As shown in our previous study [34], the hydrogen bonding between the salt (CaCl_2_) ion and meta-aramid fibers was likely responsible for the NFN structure formation.

**Figure 5:**
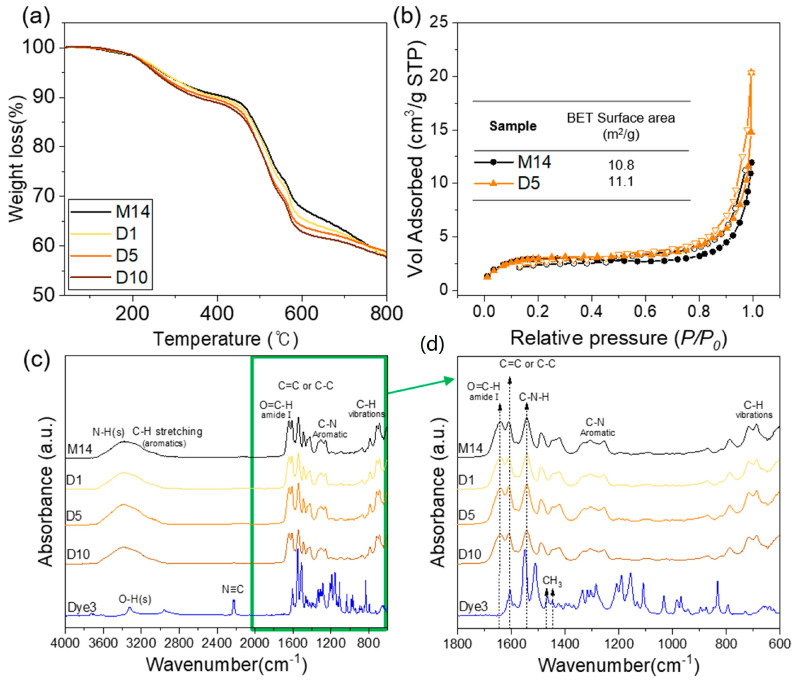
(**a**) TGA, (**b**) Brunauer–Emmett–Teller (BET) analysis of electrospun meta-aramid mats with and without dye 3, (**c**) FT-IR spectroscopy of meta-aramid nanofiber with and without dye 3 indicator, and (**d**) FT-IR spectra in the range of 1800–600 cm^−1^.

Figure 3a represents the thermal stability of the meta-aramid/dye 3 nanofiber specimens with various dye 3 concentrations. The 99% retention temperature of each nanofiber mat was approximately 162–179 °C, and it indicated the evaporation of water or residual solvent from the nanofiber mats. The temperature at 10% weight loss of pristine meta-aramid nanofiber specimens was 422 °C, and it decreased to 353 °C after adding dye 3. The thermal stability of meta-aramid/dye 3 compositions decreased gradually with increasing dye 3 content. However, the major thermal decomposition of all samples occurred at 430 °C, which was attributed to the destruction of intra- and inter-molecular hydrogen bonds and a breakdown of crystallite structure in meta-aramid fibers. The BET surface areas of both the pristine meta-aramid (M14) and meta-aramid/dye 3 (D5) nanofiber net specimens were characterized by a nitrogen adsorption–desorption isotherm, and the results are shown in Figure 5b. The BET surface area of both membranes with a porous and NFN structure was similar, approximately 10.8 and 11.1 m^2^/g, respectively, although the average diameter of the NFN structure increased after adding dye 3. To confirm the surface chemical composition, the meta-aramid/dye 3 nanofiber specimens were characterized by FT-IR. The spectrum of the pristine meta-aramid nanofiber specimen shows the absorption bands at 3415 and 1538 cm^−1^ assigned to the N-H stretch (amide II), aromatic C–C and C=C vibration at 1609 and 1489 cm^−1^, and aromatic C–N vibration at 1421 and 1307 cm^−1^ [34]. In the case of dye 3, the absorption bands at 2220 cm^−1^ were assigned to the carbon–nitrogen triple bond. The spectra of aliphatic hydrocarbon (-CH_3_, stretching) at 2957, 1466, and 1447 cm^−1^ were observed. However, a chemical interaction between the meta-aramid and dye 3 could not be verified. Considering the aromatic structures of both materials, they might have interacted via the hydrophobic effect.

**Figure 6:**
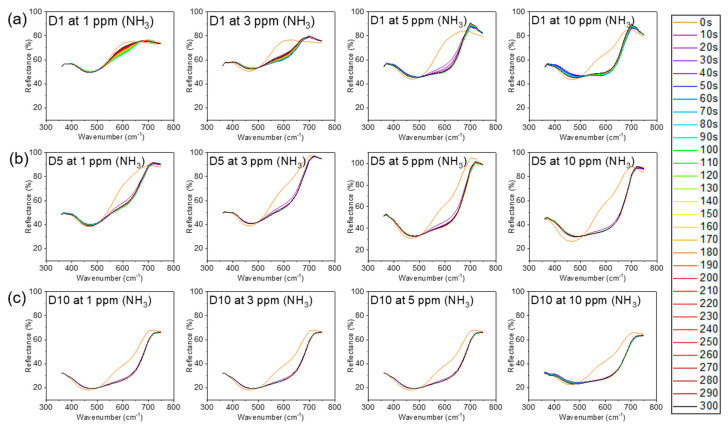
Time dependence of the reflectance of nanofibers at various NH_3_ concentrations (1, 3, 5, and 10 ppm): (**a**) dye 1 wt %, (**b**) dye 5 wt %, and (**c**) dye 10 wt %.

**Figure 7:**
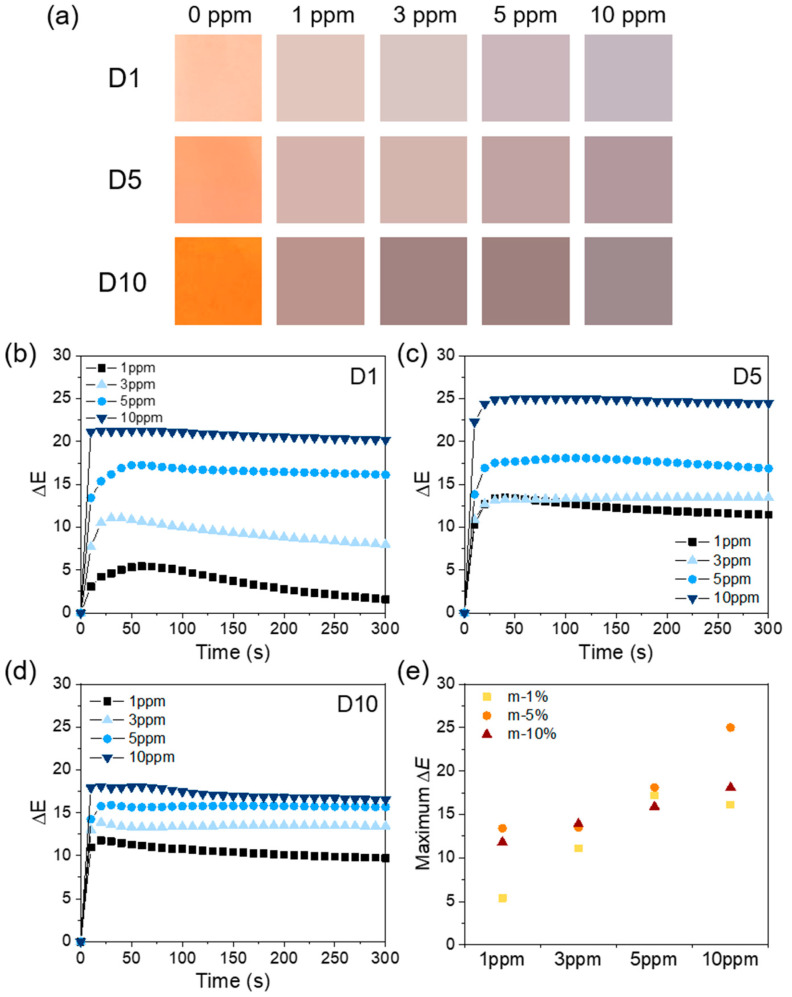
(**a**) Color changes of each nanofiber sensor after exposure to various NH_3_ concentrations (1, 3, 5, and 10 ppm); (**b**–**d**) the time-dependent ΔE values of the meta-aramid/dye 3 nanofiber sensor specimens after exposure to various NH_3_ concentrations (1, 3, 5, and 10 ppm); and (**e**) the maximum ΔE values of the meta-aramid/dye 3 nanofiber specimens containing various amounts of dye 3.

Figure 6 shows the time-dependent reflectance of the meta-aramid/dye 3 nanofiber sensor specimens under the various concentration of ammonia gas (1, 3, 5, and 10 ppm). The reflectance and L*a*b* values of the meta-aramid/dye 3 nanofiber-net sensor specimens with increasing dye 3 contents (1, 5, and 10 wt %) were measured in real time at 10 s intervals for 5 min. After exposure to NH3 gas, the reflectance of meta-aramid/dye 3 NFN at 600 nm gradually decreased. This relationship between the dye and pH can be explained by the halochromic property of the dye. When the dye 3 contacted the ammonia gas, the deprotonation of dye 3 shifted the absorption band bathochromically as shown in Figure 1a [32]. It was accompanied by a change in the color of the sensor from orange to dark brown (Figure 1b). From the L*a*b* results, the color difference (ΔE) value was calculated and is presented in Figure 7b–d.

The real color change in D1–D10 sensor specimens is shown in Figure 7a. Figure 7b–d shows the time-dependent ΔE values of the meta-aramid/dye 3 nanofiber sensor upon the exposure of various concentrations (1, 3, 5, and 10 ppm) of NH_3_. After the NH_3_ exposure, the bright or vivid orange color of colorimetric sensor specimens changed to a dull and dark brown tone. At low dye contents (D1), the ΔE value tended to increase as the concentration of gas exposure increased. Although the dye content was lower, the reaction time of D1 nanofiber sensor (reaction time = ΔE > 5) is mostly within 10 s of the NH_3_ exposure (at 3 ppm). However, when continuously exposed, the ΔE value reached a maximum and then it decreased. This could be due to the reversible reaction of NH_3_ and dye 3 or the rapid diffusion and migration of NH_3_ at low concentrations. When the amount of dye was 5 wt % (D5), the ΔE value significantly increased (25). The ΔE value also increased with increasing concentration of NH_3_. The reaction rate of D5 nanofiber sensor was faster than D1 even at low concentrations. In the case of D10, the trends in ΔE were similar to those in D5. Figure 7d shows the maximum ΔE value of each specimen upon exposure to various concentrations of NH_3_. From these results, we confirmed that the all specimens showed a distinguishable value of ΔE (>5). It could be increased by increasing either the amount of dye or the concentration of NH_3_. However, the ΔE value of D10 decreased compared to that of D5. From the FE-SEM data, we confirmed that the fiber diameter of D10 increased compared to that of D5. Therefore, the specific surface area of D10 decreased, which might have led to the inferior detection performance of the sensor. In addition, the ΔE is a relative value, which represents the degree of color change with respect to the initial state of each specimen. Therefore, although it is a good indicator of the degree of change in specimens, there is a limit to the comparison between their absolute values. From these results, we confirmed that the colorimetric green energy sensor could be employed to detect ammonia gas at concentrations 1–10 ppm with sensing response time of 10 s at room temperature.

**Figure 8:**
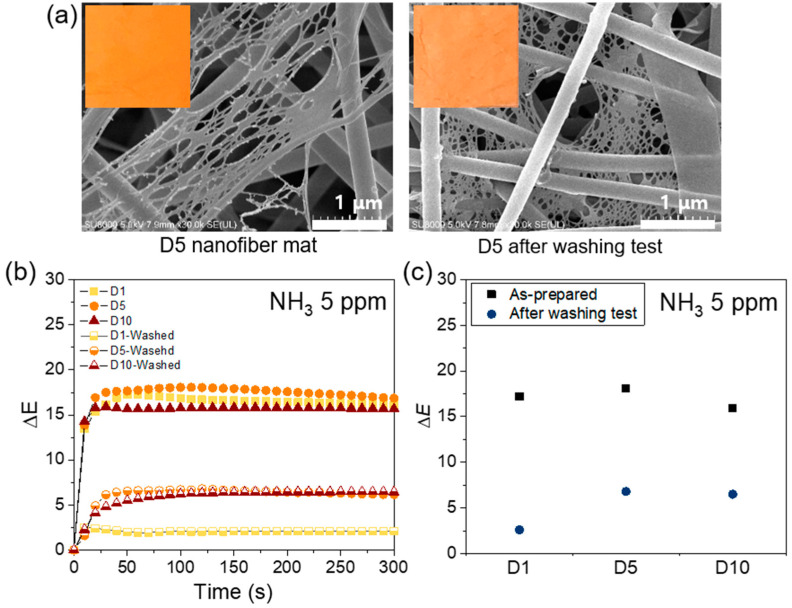
(**a**) FE-SEM image and optical image of the meta-aramid/dye 3 (D5) nanofiber before and after washing test, (**b**) the time-dependent ΔE values of the washed meta-aramid/dye 3 nanofiber sensor compared to that of as-prepared sample, and (**c**) the maximum ΔE values of the washed meta-aramid/dye 3 nanofiber compared to that of as-prepared sample.

To investigate the washability and durability of the nanofiber colorimetric sensor, the washing test was performed according to method A of standard ISO 105C10. FE-SEM images and optical photographs of a nanofiber sensor specimen before and after washing are shown in Figure 8a. There was no significant difference in the diameter of the fibers after washing; however, the net structure of the fibers appeared to merge slightly. Furthermore, it was visually confirmed that the dye 3 drained slightly from the specimen after washing. The washed nanofiber sensor specimens with various dye contents were tested with 5 ppm of NH_3_ gas and the results are shown in Figure 8b–c. After the washing test, the ΔE values of washed nanofiber sensor specimens decreased in comparison to those of as-prepared specimens. The reaction was slightly slower as well. After washing, the maximum ΔE value of D1 nanofiber sensor specimen was 2.6, which marked a decrease of approximately 84% compared to that of the as-prepared specimen. In D5 and D10 nanofiber sensor specimens, the maximum ΔE value also reduced by approximately 62% and 59%, respectively; however, these values (6.8 and 6.5, respectively) were still larger than 5, which is the detection limit of color change. These results showed that this textile-based colorimetric sensor was reusable and washable. Table 2 presents a comparison of the proposed study and the recent colorimetric nanofiber-based studies for ammonia gas detection. Table 2 confirms that the sensing abilities, such as detection limit and response time, obtained in the proposed study are comparable to those obtained in the previously reported studies. However, it is noteworthy to show that it maintained performance after standard washing.

## 4. Conclusions

In summary, our study demonstrated a facile, low cost, reusable, and washable colorimetric meta-aramid/dye 3 nanofiber sensor for ammonia (NH_3_) gas detection. The meta-aramid nanofibers were fabricated at different dye 3 concentrations; the sensor specimens exhibited a nanofiber/net structure with a high specific area. The thermal stability of the fibers decreased slightly after adding the dye; however, the fibers were relatively thermally stable compared to other polymers. The orange color of meta-aramid/dye 3 nanofiber sensor changed to dark brown due to the bathochromic shift in the absorption band of dye 3. The reaction time of meta-aramid/dye 3 nanofiber sensor (reaction time = ΔE > 5) was primarily within 10 s after the NH_3_ exposure (1 ppm). The maximum color difference (ΔE) value was exhibited by D5 at the NH_3_ exposure level of 10 ppm. After the washing test with a standard soap for 30 min, the color change in the washed fibers confirmed that the dye 3 drained slightly. However, the washed specimens still showed a distinguishable color change upon exposure to NH_3_. From these results, we confirmed the fabrication of a facile meta-aramid/dye 3 nanofiber that could be used in an ammonia gas sensor for protective clothing in industry and daily life.

## Figures and Tables

**Table 1:** Labels and basic properties of each meta-aramid/dye 3 nanofiber composition.

Label	Solution Conditions		FE-SEM		TGA ^1^		Thickness (mm)	Contact Angle (°)	Porosity (%)
	Meta-Aramid (wt %)	Dye 3 (wt %)	Fiber Diameter (nm)	Sub-Net Diameter (nm)	99%	90%			
M14	14	0	177 ± 11	21.4 ± 1.9	162	422	0.02	44.0° ± 5.1	94.9 ± 1.0
D1	14	1	238.6 ± 16	53.8 ± 27.2	169	401	0.02	94.4° ± 16.1	71.5 ± 6.1
D5	14	5	250.0 ± 25	54.2 ± 34.6	179	378	0.02	102.4° ± 11.6	84.1 ± 0.4
D10	14	10	306.5 ± 45	58.6 ± 53.3	179	353	0.02	112.0° ± 8.2	81.2 ± 2.3

^1^ The temperature (°C) at a weight retention of 99 wt % and 90 wt %.

**Table 2:** List of nanofiber-based colorimetric sensor for the NH_3_ detection.

Type of Doping	Polymer Matrix	Functionality	Response Time	Detection Limit	Washable	Ref.
Dye-doped	Meta-aramid	Dye 3	<10 s	1 ppm	O	This work
Functionalized (co) polymers	PCL	Methyl red-chitosan	<3 s	-	-	[38]
Dye-doped	PAA	Hydrazone-tricyanofuran	-	0–750 nM	-	[39]
Functionalized (co) polymers	TEOS	Methyl Red-APTES	<1 s	100 ppm	-	[10]
Dye-coating	PAN	Bromocresol Green	<1 min	1 ppm	-	[2]
